# The Role of Macrophages During Zebrafish Injury and Tissue Regeneration Under Infectious and Non-Infectious Conditions

**DOI:** 10.3389/fimmu.2021.707824

**Published:** 2021-07-21

**Authors:** Candice Bohaud, Matt D. Johansen, Christian Jorgensen, Natacha Ipseiz, Laurent Kremer, Farida Djouad

**Affiliations:** ^1^ IRMB, Univ Montpellier, INSERM, Montpellier, France; ^2^ Centre National de la Recherche Scientifique UMR 9004, Institut de Recherche en Infectiologie de Montpellier (IRIM), Université de Montpellier, Montpellier, France; ^3^ Centre for Inflammation, Faculty of Science, Centenary Institute and University of Technology Sydney, Sydney, NSW, Australia; ^4^ Clinical Immunology and Osteoarticular Diseases Therapeutic Unit, Department of Rheumatology, CHU, Montpellier, France; ^5^ Systems Immunity Research Institute, Cardiff University, Cardiff, United Kingdom; ^6^ IRIM, INSERM, Montpellier, France

**Keywords:** zebrafish, regeneration, macrophage, infectious condition, non-infectious condition, tissue injury

## Abstract

The future of regenerative medicine relies on our understanding of the mechanistic processes that underlie tissue regeneration, highlighting the need for suitable animal models. For many years, zebrafish has been exploited as an adequate model in the field due to their very high regenerative capabilities. In this organism, regeneration of several tissues, including the caudal fin, is dependent on a robust epimorphic regenerative process, typified by the formation of a blastema, consisting of highly proliferative cells that can regenerate and completely grow the lost limb within a few days. Recent studies have also emphasized the crucial role of distinct macrophage subpopulations in tissue regeneration, contributing to the early phases of inflammation and promoting tissue repair and regeneration in late stages once inflammation is resolved. However, while most studies were conducted under non-infectious conditions, this situation does not necessarily reflect all the complexities of the interactions associated with injury often involving entry of pathogenic microorganisms. There is emerging evidence that the presence of infectious pathogens can largely influence and modulate the host immune response and the regenerative processes, which is sometimes more representative of the true complexities underlying regenerative mechanics. Herein, we present the current knowledge regarding the paths involved in the repair of non-infected and infected wounds using the zebrafish model.

## Introduction

Regenerative multicellular organisms have developed different strategies to respond to injury, resulting in vastly different regenerative processes (1). Among them, some display remarkable regenerative capabilities, sometimes conserved throughout their entire life. Invertebrates, such as planarians, can regenerate complete organs or appendages from nearly every part of their body (2). Among vertebrates, teleosts, such as the zebrafish (*Danio rerio*), have emerged as excellent models for wound healing, due to their high regenerative capacity to regrow entire limbs and tissues. In this review, we will mainly focus on zebrafish, an animal model of choice, which has been intensively studied for its regenerative properties under non-infectious conditions.

Zebrafish present many advantages for fundamental biological research. They are cheaper to maintain as compared to mice and can produce 100-200 progeny every five days, allowing robust statistical analyses. Embryos are small and optically transparent, which facilitates high resolution real-time imaging (3). Moreover, zebrafish are conducive to genetic manipulation with high performance tools, including antisense morpholino oligonucleotides, CRISPR/Cas9-gene editing or insertion of Tol2 transgenes. Importantly, the zebrafish genome shares 70% homology with the human genome, offering the possibility to study a wide panel of human diseases such as muscular dystrophies, cardiovascular diseases or infectious diseases. Zebrafish represent a widely used model to investigate regenerative mechanisms of a large diversity of tissues and organs (liver, pancreas, jaw, lateral line’s hair cells, retina, heart, central nervous system and caudal fin) throughout their various developmental stages (4–15).

Tissue damage can occur under various circumstances, including non-infectious and infectious conditions. Non-infected injuries, caused by amputation, burns, freezing, crushing or exposure to toxic drugs, represent the most often studied pathologies, encompassing all forms of tissue damage that are not associated with pathogenic microbes (16–18). These injuries lead to the rapid recruitment of immune cells to the injured area, of which macrophages (MФ) play an essential role in ensuring complete regeneration and avoiding chronic inflammation (19, 20). Infected wounds, in contrast, are infrequently studied but are sometimes more biologically relevant to wounds generated throughout life and are characterized by damage in the presence of infectious microorganisms. These include open wounds or organ injuries in direct contact with contaminated environments by pathogens. In this context, the immune system is simultaneously confronted with distinct sets of signals, emerging from the lesion and from the infection foci and/or from the pathogen itself. In this complex network of interactions, MФ plays a central role, responding to the pathogen and participating in regeneration (19, 21–23).

Although not reflecting the complex interactions associated with injury, to date, most zebrafish studies have been performed under non-infectious conditions. Therefore, improving our knowledge regarding the underlying mechanisms of regeneration requires a comprehensive view of the molecular and cellular elements participating in regeneration of non-infected and infected wounds. As such, this review aims to present the current understanding of the role of MФ in the regeneration of non-infected and infected injured tissues in the zebrafish model. It also discusses the implications of abnormal MФ-mediated cellular responses in the presence of microbial pathogens, which can lead to altered regenerative processes.

## Zebrafish As Model To Study Tissue Regeneration

Two types of regenerative processes involving a cell proliferation step have been described in the zebrafish model. These two distinct processes; the epimorphic and compensatory mode, occur during zebrafish heart regeneration (24). While compensatory regeneration involves the recruitment and proliferation of mature and differentiated cells, the epimorphic regeneration occurs through the formation of a highly proliferative mass of undifferentiated cells called the blastema. The epimorphic regeneration, mainly presented in this review, has been described in several regenerative species, such as salamanders and planarians (25, 26). Epimorphic regeneration results in the complete restoration of certain appendages after amputation by restoring their original mass, structure and function. The regenerative blastema, representing the paramount step in this process, is defined as a highly proliferative and heterogeneous structure whose cellular composition is not yet fully elucidated, albeit being intensively studied (27). Lineage tracing experiments in the adult zebrafish caudal fin following amputation unraveled the cellular diversity of the blastema, mainly composed of mesenchymal, epithelial and hematopoietic cells (28). Among the hematopoietic cells, distinct MФ subpopulations have been identified and their pivotal role in blastema formation has been demonstrated (19, 22).

While most experimental studies on regeneration in zebrafish have been formulated under non-infectious conditions, natural injury sometimes involves the entry of pathogenic microorganisms. In addition, immune cells, including MФ, which are central to the regeneration process are also effector cells involved in the response to infection. Thus, the following sections will be dedicated to zebrafish MФ and their role in the response to non-infected and infected injuries.

## Macrophages In Zebrafish Tissue Regeneration

The zebrafish innate immune system has previously been described by Herbomel and colleagues (29). At one day post-fertilization (dpf), embryos already possess functional phagocytes capable of eliminating exogenous microorganisms. Mainly composed by neutrophils and MФ, the larval innate immune system shares similar functions to the one found in mammals (30, 31). Similarly to mammalian embryos, neutrophils and MФ in zebrafish appear in successive waves, emerging first from the side plate of the mesoderm and invading the larvae from 12 to 24 hours post-fertilization (hpf), in a M-CSF dependent manner (32). Subsequently, a second wave of MФ originating from the hematopoietic stem cells (HSC) occurs, reaching its definitive site at 4 dpf. From 72 hpf onward, MФ are found in different peripheral tissues including the brain, heart, muscle and retina, while others accumate in the caudal hematopoietic tissue (CHT). To date, tissue resident MФ and circulating monocytes/MФ in zebrafish cannot be distinguished, mainly because of the lack of specific markers. Most previous studies have focused on their spatial distribution, largely relying on the optical transparency of the zebrafish embryo (33, 34). A recent study of microglial ontogeny and composition has utilized RNA sequencing analysis to identify two distinct microglial populations of resident macrophages in juveniles and adult fish (35). This work highlighted the heterogeneity of the microglia, similarly to the human brain which, importantly, is not represented in the murine model.

The generation of transgenic lines of zebrafish have allowed the identification of several MФ subtypes. Two markers are mostly used to monitor circulating and tissue resident MФ: The Macrophage Expressed Gene 1 (*mpeg1*), coding for the perforin protein (36) and the Microfibrillar Associated Protein 4 (*mfap4*) (37). In addition, to further scrutinize MФ polarization into a pro-inflammatory phenotype, recent investigations have led to the generation of transgenic lines with the GFP fluorescent marker to track the expression of tumor necrosis factor (TNF) (19, 22). A deeper characterization of zebrafish myeloid cell populations based on single cell RNA sequencing is, however, warranted as it may conduct to a more thorough description of the diversity of myeloid cells and the identification of a specific signature for each cell subset during the embryonic development.

Based on these latter myeloid cell-specific markers, the role of myeloid cells, particularly MФ, has been widely investigated in zebrafish in response to infection. The function and contribution of MФ in the control of infection largely depends on the nature of the invading pathogen. While MФ largely convey protection of zebrafish embryos infected with *Staphylococcus aureus* (23), it is the depletion of MФ which protects them against *Burkholderia cenocepacia* infection (38). In the case of infection by *Mycobacterium marinum*, MФ can either protect or damage the organism. This natural fish pathogen has been extensively studied due to its close phylogenetic relationship with *Mycobacterium tuberculosis*, the causative agent of tuberculosis (39, 40). The use of *M. marinum* in zebrafish has revealed unanticipated insights into mycobacterial pathogenesis, granuloma formation and host susceptibility, and these advances are currently being paralleled in human clinical trials (41). RNA sequencing of the pro- and anti-inflammatory MФ has been carried out in zebrafish larvae infected with *M. marinum* at the early stage of the granuloma development, revealing a switch in the infected MФ inflammatory status with an increased expression of pro-inflammatory genes like CXCL Motif Chemokine Ligand 11-aa (*cxcl11aa*) and Tumour Necrosis Factor alpha (*tnfa*), as well as a decreased expression of non-inflammatory genes like interleukin-10 (*il10*) or transforming growth factor beta-1a (*tgfb1a*) (42). This pro-inflammatory phenotype might result in MФ necrosis with subsequent bacterial expansion and dissemination. This necrotic event takes place through the increased production of Reactive Oxygen Species (ROS) and calcium (Ca^2+^) in the mitochondria, activating a mitochondrial matrix protein cyclophilin D, which promotes the opening of the mitochondrial permeability transition pores (mPTP), disturbing the membrane potential and resulting in necrosis (43–48). Despite these latter results, transient depletion of the MФ impairs the response to *M. marinum* as well as to other mycobacterial species (*Mycobacterium abscessus, Mycobacterium fortuitum* and *Mycobacterium kansasii*) (49–51), indicating that MФ are crucial to restrict the bacterial expansion and the formation of granulomas that constrain mycobacterial dissemination (52). The origin of the MФ recruited during *M. marinum* infection, located in the larval brain, has also been investigated. The microglial cells were distinguished from the macrophages arriving from the circulation thanks to the injection of Hoechst coloration into the blood stream. This dye marks all the circulating monocytes/macrophages but cannot cross the blood-brain barrier, resulting into Hoechst-negative microglial cells. These cells were the first to be recruited to the site of infection, to phagocytose the bacilli and eradicate the infection, unlike the circulating monocytes, which favored bacterial spreading (34).

## Role Of Macrophages During Regeneration Of Non-Infected Tissues

During recent years, MФ have been widely scrutinized in the context of tissue regeneration. The inflammatory mechanisms associated with the regenerative processes depend on both the tissue origin and the nature of the damage. However, to date, most experimental investigations targeting the caudal fin or heart were conducted under non-infectious conditions.

### Role of Macrophages During the Regeneration of Non-Infected Caudal Fin

**Figure 1:**
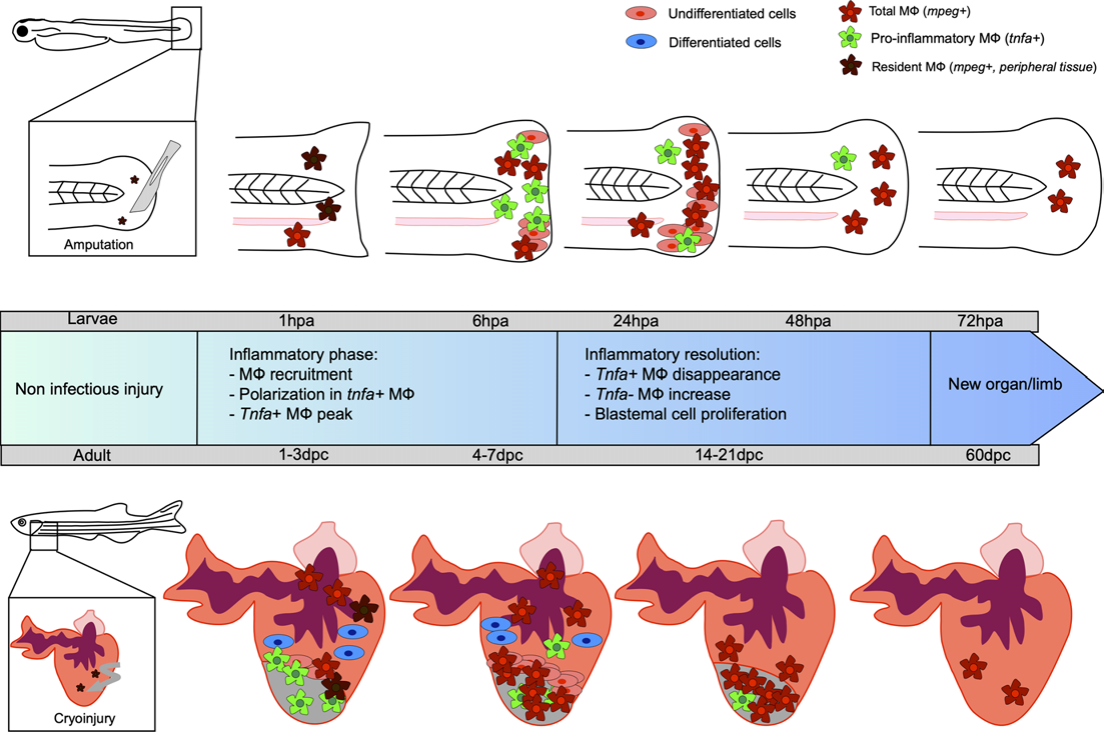
Kinetic of caudal fin and heart regeneration under non-infectious conditions. Transection of the zebrafish embryo caudal fin in non-infected condition leads to early recruitment of MФ, which complements the pool of resident MФ already present in the tail. At this very early stage, resident macrophages (MФ) phagocytose debris and dead cells. During the inflammatory phase, some MФ undergo polarization into *tnfa*-positive pro-inflammatory MФ, reaching a peak at 6 hpa. The *tnfa*-positive MФ disappear and a majority of non-inflammatory *tnfa-*negative MФ accumulate. During resolution of the inflammation, the undifferentiated blastema cells proliferate, peaking at 24 hpa, resulting in new limb formation. Cryoinjury of the heart of the adult zebrafish leads to an early recruitment of MФ, completing the pool of resident MФ already present in the heart, between 1 and 3 dpc. Tnfa-positive pro-inflammatory MФ predominantly arrive at the site and the proliferation of undifferentiated cells around the wound constituting the blastema begins. Already differentiated cardiomyocyte cells participate in tissue restoration. Non-inflammatory MФ represent the major cells present until the end of the regeneration process, resulting in a healed organ.

The caudal fin regenerates within three days after injury at the embryonic stage (at 3 dpf) and after several weeks in adults (53, 54). The growing embryonic environment as well as the structural simplicity of fin regeneration explains the fast regrowth in young larvae (55). The most commonly used model to study fin regeneration in non-infectious condition relies on amputation, although several studies have used cryoinjury and burn (56, 57). As previously mentioned, caudal fin regeneration depends on a process referred to as epimorphic regeneration, which involves three main phases resulting in blastema formation (58). A short phase of tissue repair is initiated during the first six hours post amputation, which consists of the contraction of the injured tissue and the formation of a wound epithelium. This takes place without any cell division and relies solely on the migration of epidermal cells. Epithelial remodeling is then followed by the formation of a thicker apical epithelial cap (AEC) that occurs through the secretion of matrix metalloproteinases (MMP) (59–64). The AEC plays a key role in the induction of genes required for the following steps, notably by promoting the establishment of the blastema between 12 and 48 hours after injury (65). Indeed, a tightly regulated and regionalized molecular dialogue occurs between the AEC and the underlying cells through the release of factors that include bone morphogenetic protein (BMP), the Fibroblast Growth Factor (FGF) and Wnt/β catenin pathway. The progenitor cells contained in the blastema then differentiate, giving rise to a novel caudal fin identical to the original one. Leukocytes and more specifically MФ, are essential for caudal fin regeneration (19, 20, 66). Using transgenic adult zebrafish to selectively ablate MФ, the stage-dependent functional roles of MФ in mediating the outgrowth of the fin and the morphogenesis of the bony ray partly *via* the regulation of blastemal cell proliferation was shown (66). Similarly, in zebrafish larvae, the sequential chemical depletion (lipochlodronate-induced) and genetic depletion [T*g(mpeg: Gal4; UAS: NTR-mCherry)*] treated with metronidazole) of MФ at different time points during fin fold regeneration confirmed their role during epimorphic regeneration (19). Indeed, the specific depletion of pro-inflammatory MФ inhibits the blastemal cell proliferation and blastema formation, demonstrating their importance during the early phase of regeneration. In contrast, ablation of non-inflammatory MФ leads to defective regeneration caused by an alteration of mesenchymal cells behavior without modifying cell death or the proliferation rate (19). In line with these findings, an impairment of the regenerative capability associated with an enhanced susceptibility to apoptosis of the blastemal cells has been shown in several zebrafish mutants, including *cloche* and *tal1*, characterized by a loss of most hematopoietic tissues and lacking myeloid cells (20). Going further, the authors demonstrated that blastemal cell survival and proliferation during the regeneration of the zebrafish fin was mediated by a trophic factor released by the hematopoietic cells, suggesting that blastema formation is dependent on myeloid cells (20). Similarly, when the MФ were depleted, such as in the *cloche* mutant, a dysregulated expression of *il*-1β was reported. After fin transection in zebrafish larvae, *il*-1β overexpression induces an abnormal expression of blastemal markers such as *junbl* (20). Altogether, these studies reveal that MФ allow a tight control of the inflammatory response and that the transient MФ-mediated inflammation is likely to be required for regeneration (20, 67). In this context, we showed that MФ provide a favorable inflammatory environment for the establishment of the blastema *via* the expression of paracrine factors, such as *tnf*α (19) (**Figure 1**).

Following neutrophil recruitment at the site of injury, different subpopulations of MФ are recruited, peaking at 6 hpa. The mechanisms involved in the recruitment and the activation of MФ into the wound are not yet completely understood. However, early signals induced by the amputation of the fin of zebrafish larvae regulate the recruitment of macrophages. Indeed, repressing calcium waves, the first signal induced after tissue injury, significantly impairs the recruitment of MФ at the wound site. The second signal is related to the production of ROS. The intra-MФ ROS and those generated extrinsically by the epithelium of the wound allow the early and late MФ recruitment (68). Recently, the use of H_2_O_2_ or NADPH oxidase inhibitors to modulate ROS concentrations revealed that the ROS induced by the wound are required for MФ activation but not for their recruitment (69). Despite the controversy on the role of ROS on MФ recruitment that might be due, in part, to the source and the type of ROS produced at the wound site, most studies emphasize the important role of ROS in macrophage polarization (69–71).

Using the double transgenic line *Tg (mpeg1:mCherryF/tnfa:eGFP-F)*, we recently uncovered at least two MФ subtypes displaying similarities with their mammal counterparts (22). After amputation, MФ exhibiting pro- or non-inflammatory phenotypes are rapidly recruited to the wounded site and reside there during the entire regenerative process. MФ expressing pro-inflammatory genes such as *tnfa*, interleukin-6 (*il-6*), and interleukin-1β (*il1-b*) mainly accumulate at the wounded site at 6 hpa. Parabiosis experiments combined with the use of morpholino oligonucleotides stressed the role of the TNFa/TNFR1 pathway in the establishment of the blastema by directly activating blastema cell proliferation (19). Regarding the non-inflammatory MФ expressing factors *tgf*β, *ccr*2 and *cxcr*4b, these cells were present during the entire regenerative process, peaking between 24 and 72 hpa.

With respect to the origin of MФ participating in fin regrowth after amputation, they are likely “resident” MФ in peripheral tissues (appearing with the first wave of MФ generation during early embryonic development) or/and MФ derived from the CHT. After injury and using the photoconvertible *Tg(mpeg1:dendra2)* line, it was found that peripheral MФ are recruited prior to CHT MФ and that they are more prone to respond to early stress signals, such as ROS production, and to assist the recruitment of the CHT-derived MФ. The reduced frequency of peripheral MФ leads to a reduction of blastema cell proliferation between 6 and 24 hpa, an increase in cell death, as well as an increase of ROS and IL-1β production at the wound site. Overall, these findings indicate that both MФ populations are likely to play distinct roles in caudal fin regeneration and require to be further studied (33).

### Role of Macrophages During Non-Infectious Regeneration of the Heart

The heart of the adult zebrafish is known to regenerate completely within a few weeks (72, 73). It is the first organ that forms during the embryonic development, with the appearance of myocardial and endocardial progenitor cells as early as at 5 hpf (72). The fully developed heart has a very simple structure as compared to the mammalian heart but, in both cases, it possesses an atrium and a ventricle (74). In adult mammals, a myocardial infarction leads to a deposition of fibrin and scar formation responsible for the loss of heart function. In adult zebrafish, ventricle injuries lead to the formation of a fibrin clot, which is then replaced by a well-structured and functional tissue two months after injury, which is identical to the original one (4).

Different models of cardiac injuries have been proposed. Many studies rely on the resection of a piece of the ventricle, which induces neither massive myocardial cell mortality nor fibrosis, but rather leads to the full tissue regeneration. More recently, the cryoinjury model, consisting of freezing a part of the heart, has been introduced to mimic the pathophysiological process of human myocardial infarction, characterized by rapid apoptosis of cardiomyocytes, inflammation and scar formation (75). Cryoinjury of the zebrafish heart leads to transient fibrosis and a long-lasting regeneration process, as compared to the resection protocol. This might be due to the massive number of dead cells generated after the cryoinjury (and not after ventricular resection) that is first required to be cleared (76).

Heart regeneration mechanisms in zebrafish have been intensively investigated. The regenerated myocardium was first described to mainly rely on pre-existing cardiomyocytes, undergoing de-differentiation, re-entering the cellular cycle and then migrating in the wounded area of the heart (77, 78). An accurate distribution of these mitotic cardiomyocytes was analyzed using immunodetection of phosphohistone H3 and the embryonic ventricular heavy chain myosin. A transient population of undifferentiated cardiomyocytes, located very closely to the wound, has been shown to form a structure that resembles a blastema, suggesting that the heart regeneration could also rely on epimorphic regeneration (24). However, in the myocardium, a population of mature proliferating cardiomyocytes has also been identified, suggesting that heart regeneration processes are dependent on a compensatory mechanism of regeneration. The latter consists of the growth of non-injured tissues, restoring the initial mass but not the initial shape of the organ. These two types of regeneration, epimorphic and compensatory, are both required to regain a functional heart (24). Recently, the proliferation of cardiomyocytes during the regeneration of the zebrafish heart was shown to depend on myocardial Tp53 suppression by mitogen-induced Mdm2 and Rho activity (79, 80).

Other cell types, such as immune cells, promote the transient formation of pro-fibrotic tissues and then the formation of pro-regenerative tissues allowing full cardiac regeneration (81).

MФ are massively mobilized at the injury site within minutes after neutrophil recruitment, increasing the pool of already present “resident” MФ in the uninjured heart of the zebrafish. MФ accumulate until 7 dpc, when the inflammation starts to resolve (81). Similarly, to the caudal fin regeneration process, MФ are crucial for the heart regeneration since their transient depletion after lipoclodronate injection alters the heart regeneration capacity (82, 83). After a cardiac cryoinjury, pro-inflammatory MФ expressing *tnfa* appear at the wounded site, peaking at 3 dpc. These cells promote the formation of a transient scar, between 3 and 7 dpc, with type I collagen deposition. The frequency of pro-inflammatory MФ then decreases at 7 dpc, coinciding with initiation of the scar resolution. The non-inflammatory MФ (*tnfa* ^-^) are mainly found at the lesion site from 7 dpc where they promote the resolution of inflammation and complete tissue regeneration at 60 dpc (81) (**Figure 1**). Analysis of the macrophage response during cryoinjury *versus* resection in zebrafish and in newborn and adult mice allowed the first identification of an evolutionarily conserved function of MФ that participate directly to the scar formation through collagen deposition during heart regeneration or repair, respectively (84).

## Role Of Macrophages During Regeneration Of Infected Tissues

Epimorphic regeneration in zebrafish has been poorly explored in the presence of pathogens. A few rare studies have examined injuries under infectious conditions, but they have focused primarily on neutrophils (85). To the best of our knowledge, only one study has specifically studied the cellular mechanisms associated with regeneration, the MФ response and their polarization in the presence of the bacterial pathogen, *Listeria monocytogenes* (57).

### Role of Macrophages in a Burn Model With Concomitant Infection

The recruitment of MФ and neutrophils has been studied in the context of burn-type injuries both under non-infected and infected conditions with *Pseudomonas aeruginosa*, a bacterium commonly found in patients with burns. Infection with *P. aeruginosa* is responsible for significant zebrafish larval mortality and influences the regrowth of the fin (86). Increased recruitment of neutrophils and MФ occurred between 2 and 96 hours after burn. Interleukin-6 (IL-6) receptor signaling participates in the recruitment of neutrophils, but not of MФ, in the burnt tissue under non-infected conditions. However, neutrophil recruitment was not affected in burnt and *P. aeruginosa* infected wounds in IL-6 receptor deletion zebrafish mutants. Together, these results highlight the activation of different immune cell types and molecular mechanisms under non-infected *versus* infected wounds, ultimately requiring further exploration to define these complex interactions.

### Role of Macrophages During the Regeneration of Infected Caudal Fin

**Figure 2:**
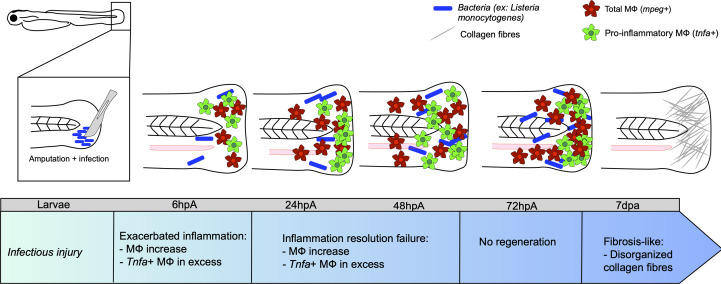
Caudal fin regeneration under infectious conditions. Amputation of the caudal fin in an infectious condition, with a scalpel pre-soaked in a solution containing pathogenic microorganisms (for instance *Listeria monocytogenes*) impairs tissue regeneration. Exacerbated inflammation is caused by an excessive number of *tnfa-*positive MФ at the wound. Resolution of inflammation is impeded at 24 hpa, with many *tnfa-*positive MФ that remain present. This results in incomplete tissue restoration and fibrosis at 7 dpa onward, characterized by the presence of disorganized collagen fibers.

The impact of infection on MФ response and polarization during caudal fin regeneration has been mainly studied after fin resection with a scalpel pre-soaked in a *L. monocytogenes* culture at 3 dpf (57). While the bacteria entered the wound and expanded, peaking at 72 to 96 hpa, they were rapidly cleared from the host by 120-168 hpa. Limited bacterial dissemination occurred along the neural tube. Under these experimental conditions, the infection did not affect larval survival, but strongly impacted regeneration even after bacterial elimination from the host. At 7 dpa, the resected caudal fin failed to regenerate and exhibited disorganized collagen fibers, characteristic of a fibrous scar. *L. monocytogenes* infection was associated with an impaired inflammatory response, characterized by higher infiltration of neutrophils and MФ at the wounded site, as compared to the non-infected transection condition. After 48 hpa, the number immune cells was very high and could not be counted individually. The pro-inflammatory response, evaluated using the Tg(*tnf:GFP*) reporter line, was exacerbated in terms of intensity and duration. Most MФ expressed *tnfa* until the end of the regeneration process (57) (**Figure 2**). Additionally, the loss of mesenchymal cells expressing vimentin, important for collagen production and fiber reorganization (87), was associated with an altered collagen reorganization (57) (**Figure 2**). However, at the end of the kinetic, at 168 hpa, the inflammation decreased, suggesting the end of the wound healing process.

## Concluding Remarks

All organisms develop strategies to respond to diseases and injuries, although these strategies greatly differ between mammals and lower vertebrates, the latter being able to completely regenerate certain organs and limbs. The zebrafish model has been increasingly adopted to improve our understanding of the underlying inflammatory response that occur during tissue and organ restoration after injury. Compelling evidence indicates that MФ play prominent roles in regeneration by releasing pro- and anti-inflammatory factors and remodeling of the extracellular matrix. They orchestrate the formation of the regenerative blastema, characterized by a population of progenitor cells capable of interacting with epithelial cells and necessary for reconstruction of the injured area. While these cells are important for homeostasis and tissue development, they are also considered as major professional phagocytes in the defense against exogenous microorganisms, leading to the hypothesis that infection influences the outcome of tissue regeneration. Listeria infection was associated with a delayed regenerative capacity in zebrafish larvae with intense inflammation and tissue damage. However, it remains to be established whether similar mechanisms are dependent on the infectious doses of Listeria and whether they are conserved with unrelated bacterial pathogens or other pathogenic microorganisms (viruses, parasites). Future studies should investigate the recruitment, activation and polarization of MФ in the presence of other pathogens in the context of an infected wound. It is anticipated that expansion of the resection/infection zebrafish models will largely contribute to increase our understanding of tissue healing and regeneration under infected conditions. Consequently, this may result in the development of new *in vivo* approaches devoted to the crosstalk between innate immunity and the remodeling machinery in response to injuries and eventually lead to promising therapies targeting the pathogen and displaying pro-resolution capabilities in complex lesions.

## Author Contributions

CB, NI, and FD structured the different sections of the review. CB, MJ, CJ, NI, LK, and FD wrote the review. All authors contributed to the article and approved the submitted version.

## Funding

We acknowledge funding support from the Inserm Institute and the University of Montpellier.

## Conflict of Interest

The authors declare that the research was conducted in the absence of any commercial or financial relationships that could be construed as a potential conflict of interest.
